# Seabirds supply nitrogen to reef-building corals on remote Pacific islets

**DOI:** 10.1038/s41598-017-03781-y

**Published:** 2017-06-16

**Authors:** Anne Lorrain, Fanny Houlbrèque, Francesca Benzoni, Lucie Barjon, Laura Tremblay-Boyer, Christophe Menkes, David P. Gillikin, Claude Payri, Hervé Jourdan, Germain Boussarie, Anouk Verheyden, Eric Vidal

**Affiliations:** 1 Institut de Recherche pour le Développement (IRD), LEMAR - UMR 6539 (UBO, CNRS, IRD, IFREMER), BP A5, 98848 Nouméa cedex, New Caledonia; 2 Laboratoire d’Excellence CORAIL, ENTROPIE (UMR9220), IRD, 98848 Nouméa cedex, New Caledonia; 3 Department of Biotechnology and Biosciences, University of Milano-Bicocca, Piazza della Scienza 2, 20126 Milan, Italy; 4 Pacific Community, Oceanic Fisheries Programme, BP D5, 98848 Nouméa, New Caledonia; 5 IRD/Sorbonne Universités (UPMC, Université Paris 06)/CNRS/MNHN, LOCEAN – UMR 7159, BP A5, 98848 Nouméa cedex, New Caledonia; 6 Department of Geology, Union College, 807 Union St., Schenectady, NY 12308 USA; 7 Institut Méditerranéen de Biodiversité et d’Écologie marine et continentale (IMBE), Aix Marseille Université, CNRS, IRD, Avignon Université, Centre IRD de Nouméa, BP A5, 98848 Nouméa cedex, New Caledonia

## Abstract

Seabirds concentrate nutrients from large marine areas on their nesting islands playing an important ecological role in nutrient transfer between marine and terrestrial ecosystems. Here we investigate the role of guano on corals reefs across scales by analyzing the stable nitrogen isotopic (δ^15^N) values of the scleractinian coral *Pocillopora damicornis* on fringing reefs around two Pacific remote islets with large seabird colonies. Marine stations closest to the seabird colonies had higher nitrate + nitrite concentrations compared to more distant stations. Coral and zooxanthellae δ^15^N values were also higher at these sites, suggesting that guano-derived nitrogen is assimilated into corals and contributes to their nitrogen requirements. The spatial extent of guano influence was however restricted to a local scale. Our results demonstrate that seabird-derived nutrients not only spread across the terrestrial ecosystem, but also affect components of the adjacent marine ecosystem. Further studies are now needed to assess if this nutrient input has a positive or negative effect for corals. Such studies on remote islets also open fresh perspectives to understand how nutrients affect coral reefs isolated from other anthropogenic stressors.

## Introduction

Ecosystems, even those with seemingly distinct borders, rarely function independently of other adjacent systems^1^. Ecologists are increasingly recognizing the important effects that cross-ecosystem transport of energy and nutrients have on plant and animal populations and communities^2, 3^. A well known example of this is how seabirds concentrate marine-derived nutrients on breeding islands in the form of feces (guano) which contains ~15–20% nitrogen (N), as well as 10% phosphorus^4–6^. These nutrients dramatically alter terrestrial ecosystem functioning and dynamics and can support increased primary and secondary productivity^7, 8^. However, although many studies have demonstrated nitrogen enrichment of terrestrial components due to guano deposition across various taxonomic groups^7, 9–11^, only a few have studied its retroaction on marine ecosystems and most of these studies were restricted to temperate regions and high nutrient waters^4, 12–14^. In the tropics, coral reefs can be found adjacent to islands with large populations of breeding seabirds, and could be potentially affected by local nutrient enrichment due to the transport of seabird-derived nutrients in surrounding waters. While two studies have explored the influence of guano on these tropical marine ecosystems^12, 15^ and suggested that nitrogen from guano enriched seawater and reef primary producers, none have focused on its impact upon corals.

Reef building corals have essential nitrogen needs and, thriving in nutrient-poor tropical waters^16^ where nitrogen is a major limiting nutrient for primary productivity^17^, they have developed specific adaptations for conserving this element. Their establishment and maintenance are partly due to their symbiosis with unicellular dinoflagellates, *Symbiodinium spp*. (zooxanthellae), that can take up and retain dissolved inorganic nitrogen (ammonium and nitrate) from the surrounding waters^18–20^. These zooxanthellae can also recycle the animal wastes and subsequently transfer them back to the coral host as amino acids^21^, ammonium or urea^22^. Corals are also able to ingest nitrogen-rich sediment particles^23, 24^ and plankton^25, 26^. While it is widely admitted that coastal eutrophication and excess nutrient supply have a strong impact on corals, leading essentially to a decrease in skeletal growth^19, 27–30^, the potential effects of nutrients from the breeding sites of seabirds on coral reefs has never been studied.

**Figure 1:**
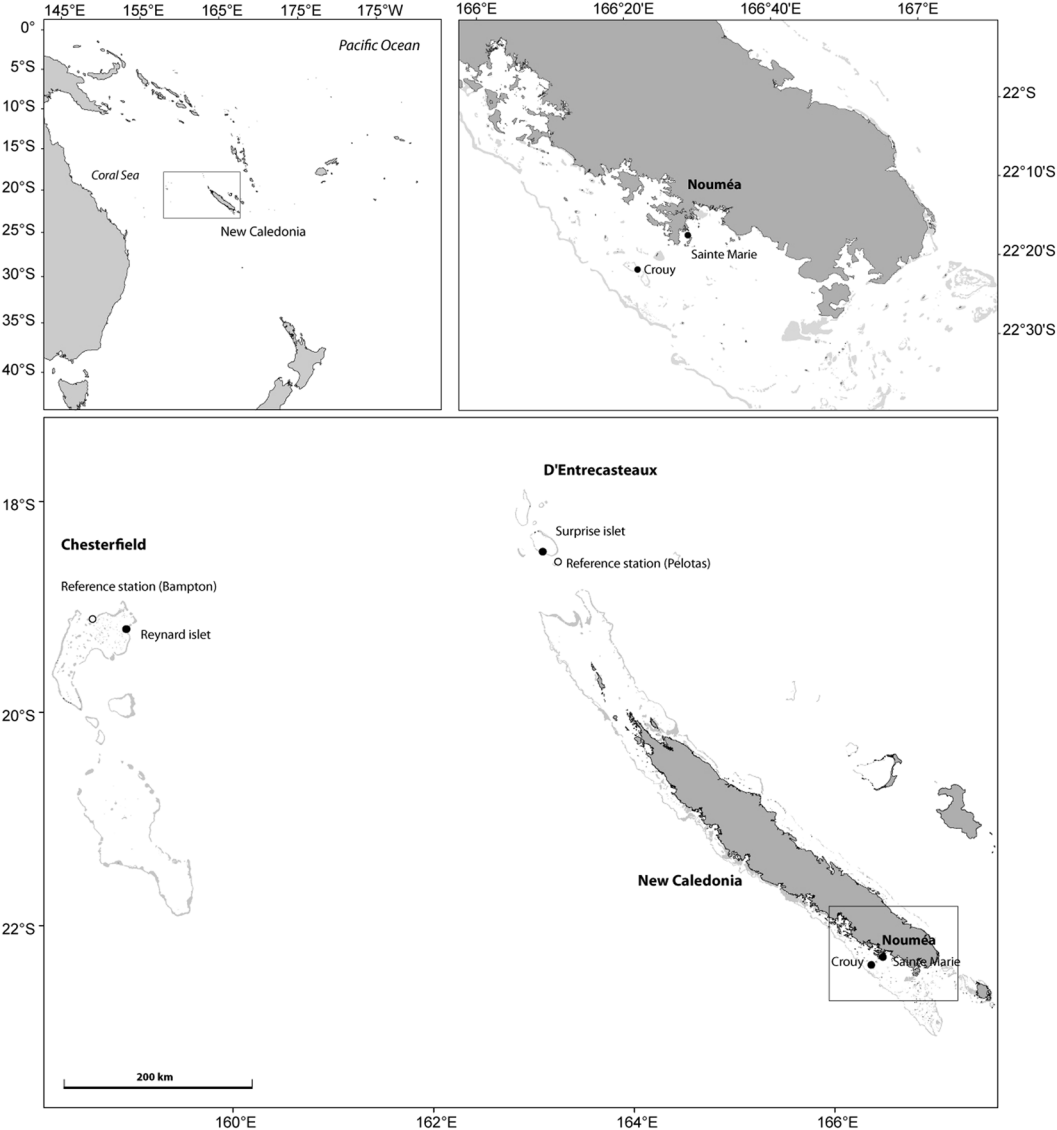
Sampling sites in New Caledonia. The map was generated with ArcGIS 10.2.2 (http://www.esri.com/arcgis/aboutarcgis) and customized in Adobe Illustrator CS3 (version 13, www.adobe.com).

The Chesterfield islands and D’Entrecasteaux Reefs (Coral Sea, Western Pacific Ocean) are remote reef complexes located at 550 and 230 km respectively from the New Caledonia main land^31^ (Fig. 1); they are uninhabited and isolated from anthropogenic nitrogen influences and their surrounding seawaters have typically low nutrient concentrations^32^. Both reef systems have been listed as conservation priority sites for New Caledonia and are part of the Natural Marine Park of the Coral Sea spanning 1.3 million km^2^ of marine ecosystems^33^. Reynard and Surprise, two islets in Chesterfield and D’Entrecasteaux Reefs, respectively, provide refuge for large seabird colonies (up to 40,000 breeding pairs)^34, 35^ and represent an ideal study system to evaluate seabird guano influence on adjacent coral reefs without any anthropogenic impacts. Seabird guano is enriched in ^15^N relative to ^14^N, partly due to the birds’ high trophic position^36^ and to preferential volatilization of ^14^N from guano^37^. Thus, nitrogen stable isotope ratios can be used as a useful tool to trace guano incorporation in marine food webs^7, 12^, with high δ^15^N values in animal tissues being a proxy for seabird derived nitrogen.

**Figure 2:**
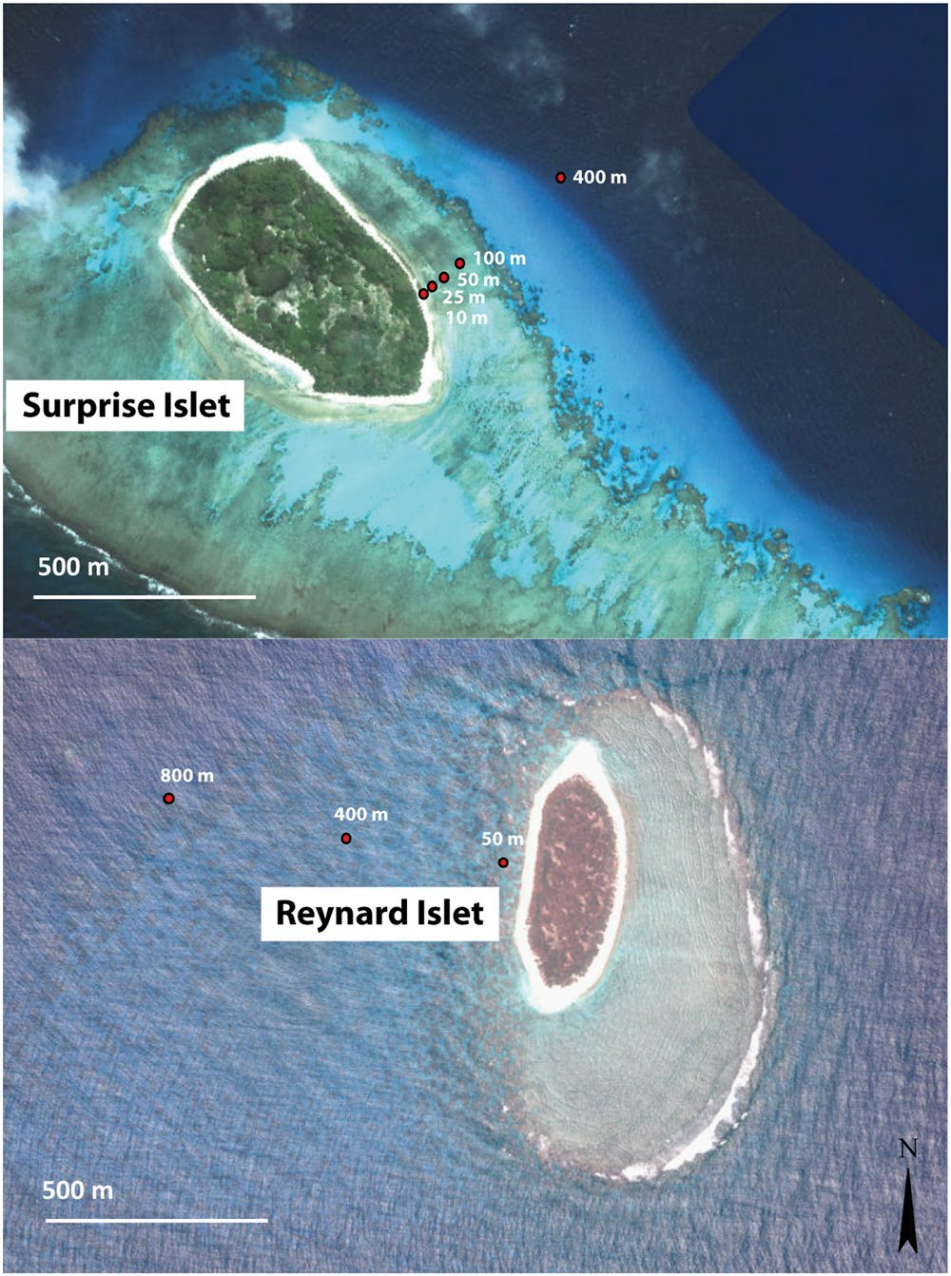
Geographic location of coral and nutrient sampling stations for Surprise and Reynard islets. The satellite images for the map were obtained from Government of New Caledonia (via the opensource portal https://explorateur-carto.georep.nc), and site locations were overlaid in Adobe Illustrator CS3 (version 13, www.adobe.com).

The main aim of this study was to assess if seabird guano was a potential nitrogen source for reef building corals and to evaluate the spatial extent of such influence on adjacent coral reefs. We selected reefs adjacent to two islets in the Chesterfield Islands and D’Entrecasteaux Reefs with thousands of breeding seabirds^34, 35^ and compared them to a reference site without nesting seabirds to assess whether seabird presence was associated with higher concentrations of nutrients (phosphate and nitrate) and higher coral δ^15^N values. *Pocillopora damicornis* is a common and locally abundant zooxanthellate scleractinian coral in the Chesterfield and D’Entrecasteaux Reefs^38, 39^. It was thus targeted for sampling to perform nitrogen stable isotope analyses in the animal and zooxanthellae tissue separately, as both compartments use nitrogen^40^. Guano was sampled from different seabirds on their nests, and seawater and coral samples were collected along a gradient from the shore (from 10 to 800 m, Fig. 2). We hypothesized that coral reef waters closest to seabird colonies would have highest concentrations of dissolved nutrients and that corals would have higher δ^15^N values compared to the farthest and reference sites. Results were also compared to those from a site subjected to high levels of anthropogenic inputs in the southern lagoon of New Caledonia (Nouméa, Fig. 1) to evaluate the importance of seabird-derived vs. anthropogenic-derived nitrogen on corals and their surrounding waters.

## Results

### Nutrients

**Figure 3:**
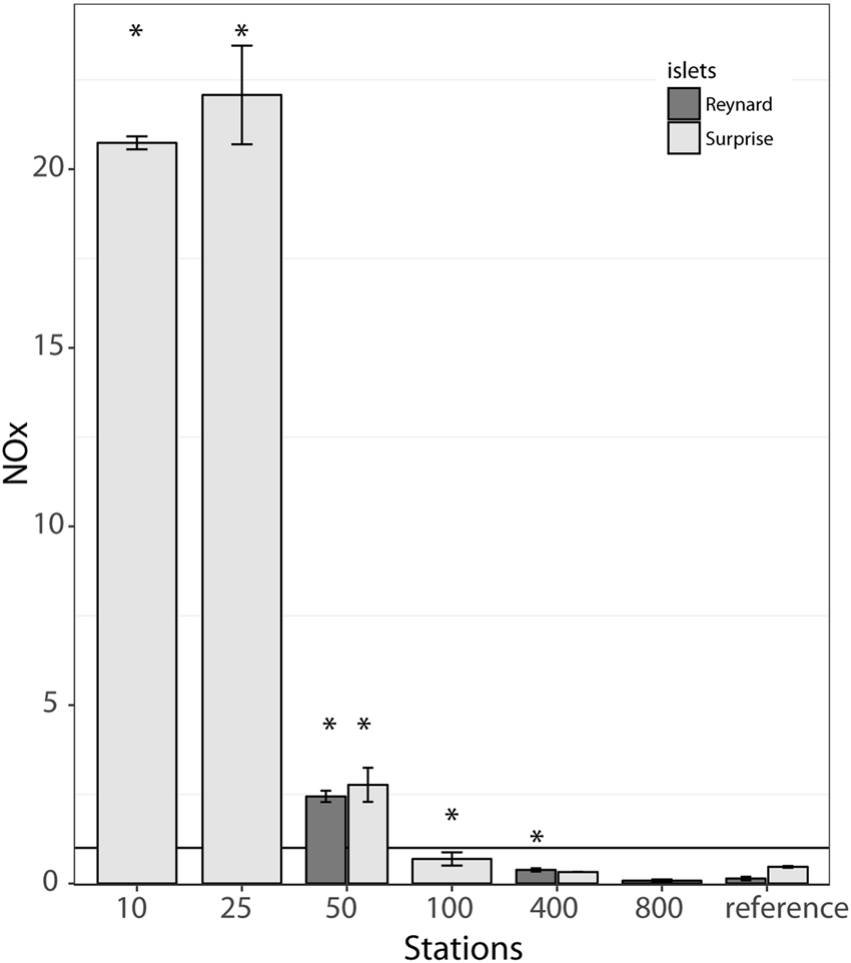
NOx concentrations (nitrates + nitrites) from Chesterfield (Reynard islet) and D’Entrecasteaux reefs (Surprise islet) in µmol/L. The black line indicates the average NOx concentration at Sainte Marie (Nouméa), a station with high anthropogenic inputs. Stars denote significantly higher values compared to the reference station at a 0.05 significance level.

At both islets, a clear NOx (nitrate + nitrite) spatial gradient was observed, with higher NOx concentrations closer to the shore (Fig. 3). At Reynard islet, we found significantly higher NOx values at 50 m and 400 m from the shore (2.44 µM and 0.38 µM, respectively) compared to the reference station (0.14 µM, Table 1 and Supplementary Table S1). NOx concentrations at 800 m were not significantly different from the reference station. At Surprise, NOx concentrations were similar and high at 10 and 25 m (~21 µM, Table 1 and Supplementary Table S1), then decreased to 2.76 µM at 50 m and 0.69 µM at 100 m. NOx concentrations were significantly higher from the reference station at all sites but 400 m. Comparing islets, NOx concentrations at 50 m (2.44 vs. 2.76 µM) and 400 m (0.38 vs 0.33 µM) were similar at Reynard and Surprise, respectively. In Nouméa, NOx values at Sainte Marie station were 0.93 µM and 0.10 µM at Crouy (Table 1).

**Table 1:** NOx (nitrates + nitrites) at the different stations in Nouméa and at Reynard and Surprise (Mean ± SD, N = 3 per station).

Date	Site	Distance from the shore	NO_x_ (µmol.L^−1^)
10/11/2015	Reynard	50 m	2.44 ± 0.13
10/11/2015	Reynard	400 m	0.38 ± 0.04
10/11/2015	Reynard	800 m	0.08 ± 0.03
22/11/2015	Bampton	Reference station	0.14 ± 0.04
05/10/2016	Surprise	10 m	20.74 ± 0.18
05/10/2016	Surprise	25 m	22.08 ± 1.38
05/10/2016	Surprise	50 m	2.76 ± 0.48
05/10/2016	Surprise	100 m	0.69 ± 0.18
05/10/2016	Surprise	400 m	0.33 ± 0.01
02/03/2017	Pelotas	Reference station	0.47 ± 0.02
31/10/2014	Sainte Marie	urbanized	0.93 ± 0.25
31/10/2014	Crouy	Reference station	0.10 ± 0.02

Reference stations were sampled at 50 m from the reef crest.

### Stable isotopes in corals and guano

Guano δ^15^N values at Reynard islet were 10.3 ± 0.3‰, 11.4 ± 0.1‰ and 18.3 ± 1.3‰ for the red-footed booby, the great frigatebird and the brown booby, respectively (N = 3 per seabird species). At Surprise islet, they were 11.5 ± 1.2‰ and 11.5 ± 0.8‰ for the red-footed booby and the great frigatebird, respectively (N = 3 per species).

**Figure 4:**
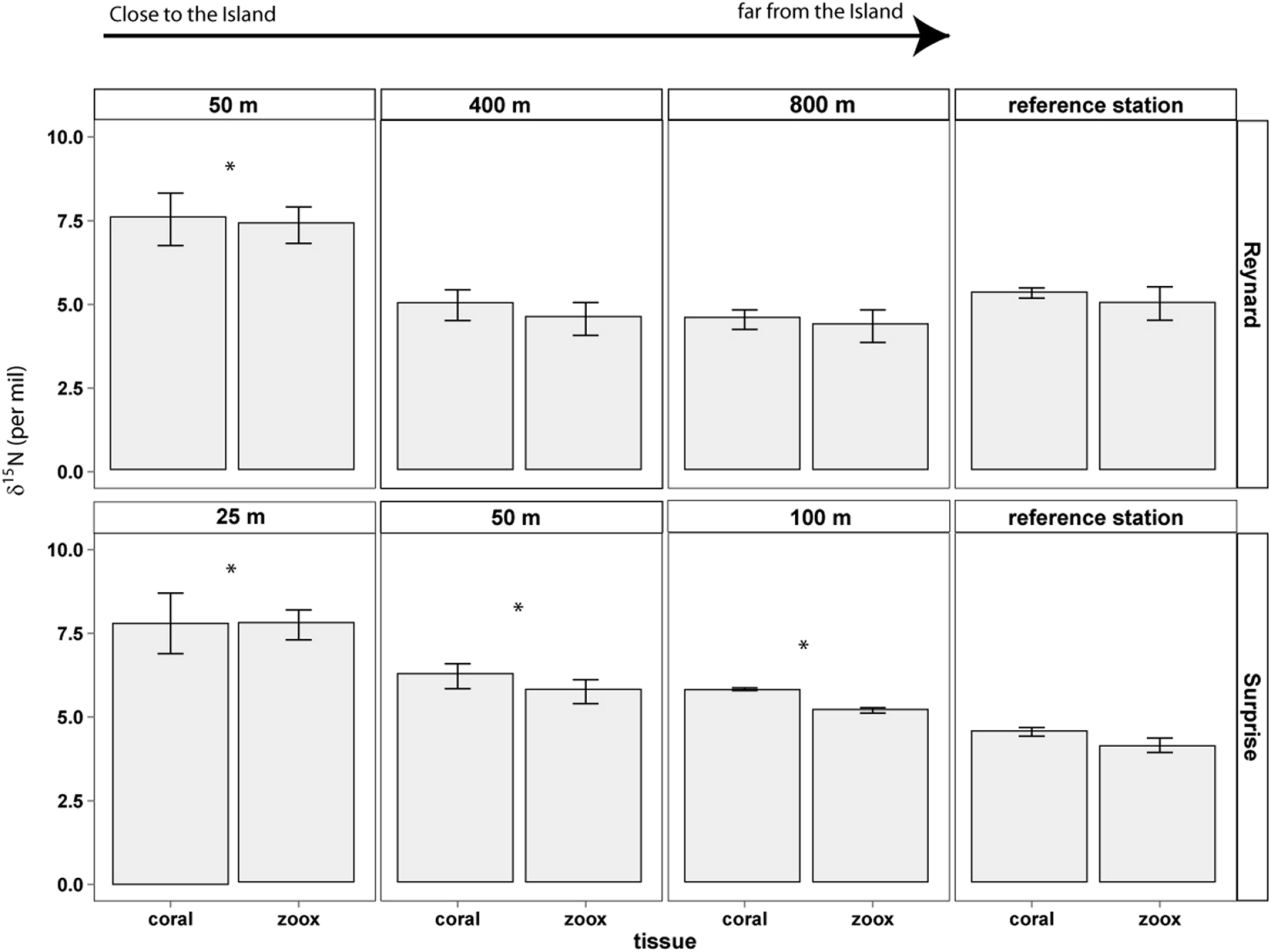
δ^15^N values from Reynard islet and Surprise islets in coral tissues and zooxanthellae. Stars denote significantly higher values compared to the reference station (0.05 significance level, statistical results for coral and zooxanthellae are the same). Note that the distances of sampled sites from the shore are different between islets.

Coral tissues and zooxanthellae showed similar spatial patterns with significantly higher δ^15^N values at the closest sites (25 m, 50 m and, when applicable, 100 m from the shore at Surprise and Reynard islets, Fig. 4 and Supplementary Table S2) compared to the reference stations. Coral tissue and zooxanthellae δ^15^N values decreased with increasing distance from the shore. More specifically, at Reynard islet, δ^15^N values in coral tissue and zooxanthellae were significantly higher at the 50 m site compared to the reference station (p < 0.001). Stations at 400 and 800 m were either significantly lower or showed no difference from the reference station. At Surprise islet, the 25 m, 50 m and 100 m sites were all significantly higher than the reference station (p < 0.001), with the 25 m showing the highest δ^15^N values for both coral tissue and zooxanthellae. When comparing the values at the two islets at matching 50 m stations, higher values were observed at Reynard islet for both coral and zooxanthellae (Table 2).

**Table 2:** δ^15^N values (mean ± SD, N = 5) in coral tissue (coral) and zooxanthellae (zoox) at reference stations (50 m from the reef crest), Reynard and Surprise islets.

site	sampling distance (m)	tissue	δ^15^N (‰)
Reynard	50	coral	7.5 ± 0.8
Reynard	50	zoox	7.4 ± 0.5
Reynard	400	coral	5.0 ± 0.5
Reynard	400	zoox	4.6 ± 0.5
Reynard	800	coral	4.5 ± 0.3
Reynard	800	zoox	4.3 ± 0.5
Bampton	Reference station	coral	5.3 ± 0.2
Bampton	Reference station	zoox	5.0 ± 0.5
Surprise	25	coral	7.8 ± 0.9
Surprise	25	zoox	7.8 ± 0.4
Surprise	50	coral	6.2 ± 0.4
Surprise	50	zoox	5.8 ± 0.4
Surprise	100	coral	5.7 ± 0.0
Surprise	100	zoox	5.2 ± 0.1
Pelotas	Reference station	coral	4.5 ± 0.1
Pelotas	Reference station	zoox	4.1 ± 0.2
Sainte Marie	urbanized	coral	5.7 ± 0.3
Sainte Marie	urbanized	zoox	4.8 ± 0.3
Crouy	Reference station	coral	3.7 ± 0.1
Crouy	Reference station	zoox	2.7 ± 0.4

Values in Nouméa stations (Sainte Marie and Crouy) are also indicated.

In Nouméa, at the Sainte Marie station, δ^15^N values in coral tissues were 5.7 ± 0.3‰ and 4.8 ± 0.3‰ in zooxanthellae (Table 2). At Crouy, far from urbanized areas, δ^15^N values were 3.7 ± 0.1‰ and 2.7 ± 0.4‰ in coral and zooxanthellae, respectively.

## Discussion

The high δ^15^N values found in both zooxanthellae and tissues of corals collected close to the seabird colonies suggest that guano is a significant source of nitrogen for them. The high coral δ^15^N values and high nutrient concentrations at sites closest to shore provide further evidence that seabirds can influence coral reefs via cross-ecosystem nutrient subsidies. However, the spatial extent of seabird influence seemed restricted to reefs close to the island within 100 to 400 m from the shore.

### Guano-derived nitrogen and coral nutrition

Seabird guano showed high δ^15^N values (on average 11‰ for the red-footed booby and the great frigatebird and 18‰ for the brown booby), in the range of values found by other studies^14, 41, 42^. These δ^15^N values not only depend on the trophic level or the diet of the seabird but also on the state of mineralization of the guano (e.g., ammonia volatilization), which can have large isotope effects and increase its isotopic values^43^. Although we have not measured the δ^15^N values of dissolved inorganic nitrogen (hereafter DIN) or particulate organic matter in the surrounding waters, we have found significantly higher NOx concentrations at sites close to seabird colonies compared to more distant sites. This guano has most probably ^15^N-enriched the base of the marine ecosystem (both dissolved and particulate phases) and caused ^15^N enrichment in the coral tissues. Such ^15^N enrichment is indeed reflected in corals as δ^15^N values found in coral tissues and zooxanthellae sampled close to the seabird colonies were ~2.5 to 3‰ higher than at farthest sites and reference sites (~8‰ vs. 5‰). This result, together with the high NOx concentrations in seawater at the examined sites, demonstrates significant local enrichment in nitrogen from seabird guano and, most importantly, uptake and assimilation of this seabird-derived nitrogen in coral tissues and their symbiotic zooxanthellae. Corals use multiple ways to supply their nitrogen needs: they can efficiently take up and retain DIN (such as ammonium and nitrate^44–46^), dissolved organic nitrogen (DON, under the form of amino acids and even urea^21, 47^), organic matter and planktonic preys^40^. Several possibilities might thus explain this coral ^15^N enrichment but our study did not allow us to determine which pathway was predominant.

To estimate the importance of guano derived N to these corals we use a simple mathematical mixing model to determine the proportions of various sources in a mixture (a common approach in isotope geochemistry^48–50^). Using such simple mixing model (%N_guano_ = (δ^15^N_nearshore coral_ − δ^15^N_reference coral_)/(δ^15^N_guano_ − δ^15^N_reference coral_) × 100) we estimate coral tissues obtain 15 to 50% of their N from seabird guano in these near-shore ecosystems. Using the reference site coral tissue and seabird guano as end-members, with no trophic enrichment from guano to coral tissue (i.e., direct uptake from DIN/DON) we calculate ~45% of N is derived from guano in near-shore corals using 11‰ as the guano value, and ~20% when using 18‰ as the guano value (i.e., range of guano δ^15^N values measured in this study). If, instead, the assimilation occurs via the planktonic preys, these values fall to about 30% and 15%, respectively, when using a traditional 3.4‰ trophic enrichment. While the end-members used here are rough approximations (i.e., guano likely undergoes several transformations before corals utilize guano derived N), it does provide a first assessment of the importance of guano derived N in these coral communities.

Such assimilation of guano-derived nitrogen in tropical waters has already been observed in macroalgae around Hawaii^12^ with δ^15^N values 1.5‰ higher in macroalgae at high seabird density sites compared to low seabird density sites, but our study provides the first evidence of its assimilation by a coral, both in tissue and its zooxanthellae. These nutrients will probably impact higher trophic level consumers such as parrot fish feeding on corals^51, 52^ and even higher trophic level organisms, since baseline isotopic values have been shown to propagate up to top predators in marine and terrestrial systems^7, 53^. Assimilation of seabird-derived nitrogen by coral and zooxanthellae could have important unanticipated effects on coral health even if it is still subject to debate whether high levels of nitrogen are beneficial or not to corals^54^. High levels of added nutrients (up to 15 µM of NH4^+^) have been shown to have direct toxic effects on corals by hindering calcification^19, 27–30^ and reducing their resistance to thermal stress^55, 56^. These studies suggest that enhanced DIN availability would promote excessive zooxanthellae growth rates, which ultimately disrupts the stability and functioning of the coral-zooxanthellae endosymbiosis^55, 57^. The combination of elevated temperature and nitrate enrichment would produce an even more pronounced reduction of coral photosynthetic production^56^. However, other studies showed that an addition of nitrates could increase coral resilience to bleaching by increasing the potential for energy storage within the coral^58–60^. Assimilation of seabird-derived nitrogen by coral could then be particularly significant in terms of resilience to climate change, especially as some studies predict that increasing sea surface temperatures will decrease the availability of nitrates^61^.

Further studies are then needed to investigate if the nitrogen enrichment from seabird guano would positively or negatively affect coral metabolism (e.g., photosynthetic efficiency, calcification rates) and their potential to recover from bleaching. Nutrient enrichment can also directly or indirectly mediate changes in community structure (e.g., from coral- to algal-dominated)^62–65^ and it would be interesting to assess such changes in these remote environments where the effect of nutrient inputs are not confounded by multiple anthropogenic stressors.

### Spatial and temporal influence of seabird-derived guano on corals and seawater

**Figure 5:**
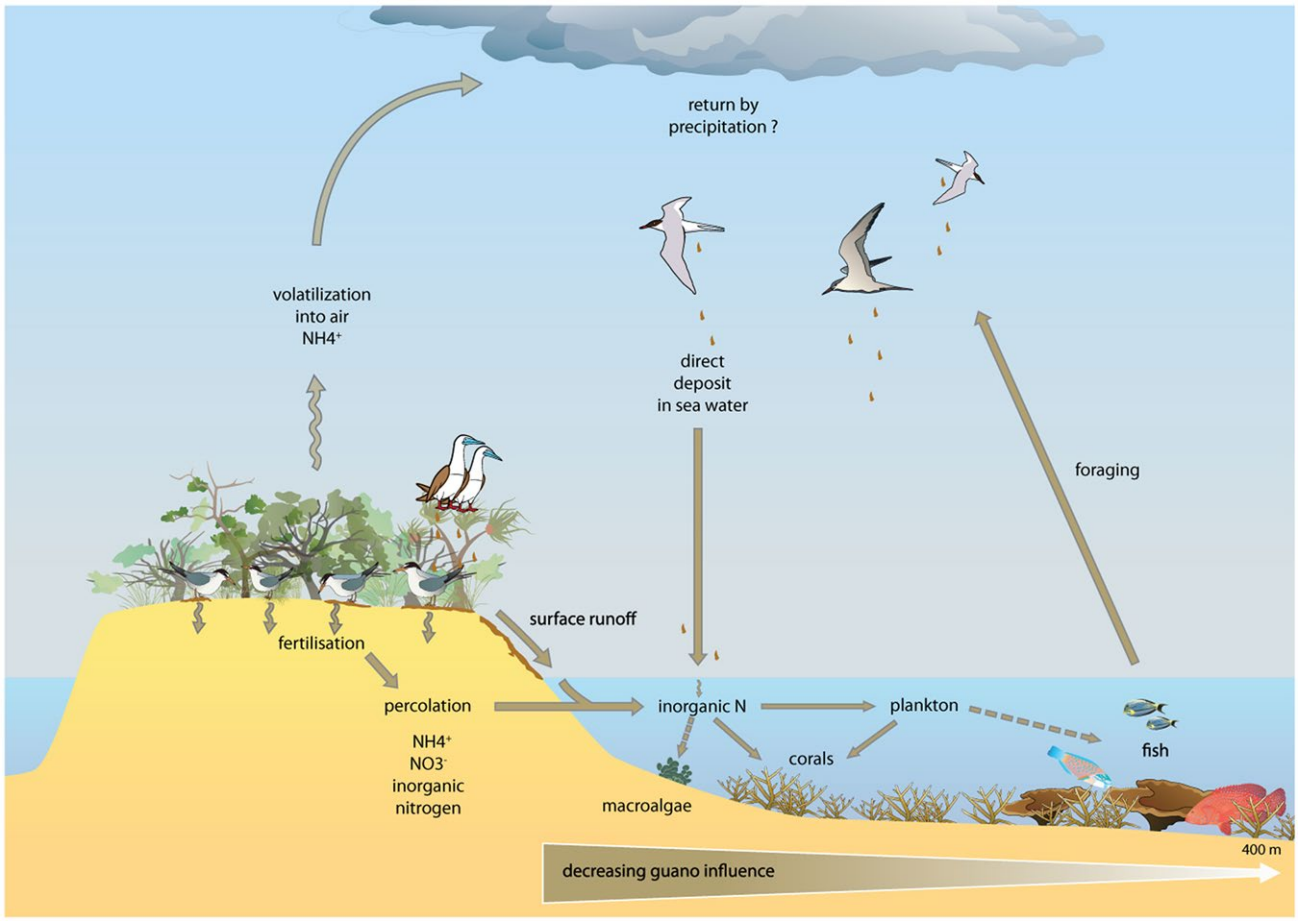
Conceptual diagram showing the different pathways for guano-derived nitrogen to enter seawater and marine food webs (brown arrows) with hypothetical transfers to fish and macroalgae in broken lines, generated with Adobe illustrator CS3 (version 13, www.adobe.com).

The influence of seabird guano on corals and their surrounding seawaters can occur through both percolation of accumulated guano on the islands via precipitation, and direct guano excretion into the water during foraging flights and return to islands^15, 66^ (Fig. 5). Increased nutrient concentrations (NH_4_^+^, NO_3_^−^, NO_2_^−^ and PO_4_^−^) in adjacent waters have already been reported from seabird islands in marine waters^12, 15^. However, compared to other studies^4, 12^, the advantage of these remote Pacific low carbonate islets as models is that the observed stable isotope enrichment is not masked by anthropogenic eutrophication. Surprise and Reynard islets, at 230 and 550 km North West of the New Caledonia mainland, respectively, are far from anthropogenic inputs, such that the observed enrichment can only be due to the transport of seabird guano into surrounding waters. As a comparison, waters in the south-west lagoon of New Caledonia were in general below 0.04 µM for NOx with values up to 1.6 µM at urbanized embayment stations^67–69^ where nutrient loading from the city is known to be elevated^68, 69^.

Local nutrient enrichment from seabird guano at remote islets can then be quite substantial, since NOx concentrations at Surprise and Reynard were much higher even than the ones found in the Sainte-Marie urban site close to Nouméa city (×10 at 10 m and 25 m, and ×2 at 50 m). Coral δ^15^N values were also higher by 2 to 3‰ compared to Saint-Marie corals, where nutrient loading from Nouméa city is known to be elevated^68, 69^. The comparison of those absolute values remains however, not very informative given the drastically different origin for the nitrogen pollution. Knowledge of the δ^15^N isotopic value of the base of the food web (particulate organic matter or DIN) would be required for an in-depth comparison.

Guano deposition could potentially have far-reaching effects^70^. Our results, however, showed the markedly localized effect of isotopic enrichment by birds, with significant differences in isotopic nitrogen values and NOx concentrations recorded at sites only 25–400 m distant from the shore. At 400 m from the shore, coral and zooxanthellae δ^15^N values were similar to the reference sites, but NOx concentrations were still higher than the reference site, suggesting that guano could still have an influence there. Therefore, while we cannot exactly assess the extent of this spatial influence, our study suggests that seawater enrichment from seabird guano might be limited to ~100 to 400 m from the shore.

The nutrient concentrations measured in our study only provide a snapshot in time in contrast to isotopic results which capture a longer time period. Coral tissue turnover rates are not known, but their tissues are unlikely to integrate more than a few months of data. For example, bivalve muscle turnover is expected to be 3 to 4 months^71^. Since our results were collected during a single season, the austral summer, we cannot rule out that the extent of enrichment may vary seasonally due to, for instance, seasonal changes in guano accumulation or rainfall. However, while a portion of the seabird species in the Chesterfield area (namely, wedge-tailed shearwater, black and brown noddies, and black-naped terns) breed in the summer, as do most seabird species south of the Coral Sea^35^, other species (masked booby, red-footed booby, frigatebirds, and fairy tern) breed in the winter^34^. Therefore, guano deposition should occur all year round at both islands, as is often the case in tropical ecosystems^72^ and its influence on coral reefs should not be restricted to a particular time or season of the year. It should be noted, however, that additional δ^15^N variability could be introduced if levels of guano deposition and ^15^N enrichment vary amongst the different seabird species that co-occur in this area. Studies at different time of the year on corals or sequential sampling of the organic matter embedded in the skeleton of bivalve shells^73^ or corals^74^ would allow to further assess the temporal importance of this cross-ecosystem nutrient exchange.

Nutrient concentration in waters surrounding seabird islands might depend on nest density, seabird species but also on island structure and vegetation, wave exposure, and weather conditions^13, 72^. The comparison between sites is difficult in our study as different distances from the shore were selected for the sampling in Reynard and Surprise islets. This comparison can only be done at 50 m from the shore with significantly higher coral and zooxanthellae δ^15^N values (~1.5‰ higher), but similar nutrient concentrations at Reynard compared to Surprise islet. This would suggest higher importance of guano-derived nitrogen on corals at Reynard islet. This result might be expected as the seabird colony is larger at Reynard compared to Surprise (40,000 vs. 5,000 breeding pairs^34, 35^) and on a smaller surface area (8 vs. 24 ha). Surprise also has more complex and stratified vegetation cover, which could impede guano spreading into surrounding waters, as well as promote N uptake by the vegetation. Further analyses with similar sampled gradients between the two islets would be needed to confirm this pattern.

## Conclusion

Our study suggests that seabird guano can be a significant source of nitrogen for corals on remote islets. To further test the importance of guano on coral nutrition, it would be interesting to analyze carbon, nitrogen and phosphorus concentrations of coral and zooxanthellae tissues to determine if seabird guano also represents a non-negligible source of phosphorus for corals, and to confirm higher nitrogen incorporation at stations close to seabird colonies. Seabirds, obtaining their food from the ocean but exerting their reproductive activities on islands, would thus not only be important vectors of nitrogen to terrestrial systems but also to the near-shore marine ecosystem. The effects found in this study are spatially restricted to reefs close to the island within ~100 to 400 m from the shore, which, to the best of our knowledge, is the first estimate for the spatial extent of guano influence on corals.

As it is widely known that corals, which regularly experience elevated concentrations of dissolved inorganic nitrogen, are undergoing important physiological modifications, it is essential to determine whether this guano input also affect their physiological state and their resistance to climate change. Further studies on isotopic variations over time and space in a broader range of taxonomic groups (e.g., invertebrates, fish, and macroalgae) would contribute to our understanding of the seabirds’ role in near-shore ecosystem communities. This is particularly true in a context of global change and the accompanying shifts in seabird populations worldwide^75–77^. Finally, given the challenges of studying the impact of nutrients on corals in isolation of other anthropogenic stressors (e.g., heavy metals, human eutrophication), remote islets with nesting seabird populations provide an ideal study system to investigate how nutrient enrichment in general can affect marine environments.

## Materials and Methods

### Study sites

This study was conducted at two remote coral reef islets in the Coral Sea (South Pacific, New-Caledonia): Surprise in the D’Entrecasteaux Reefs, and Reynard in the Chesterfield Islands (Fig. 1). Surprise Islet is part of the Surprise atoll located 230 km north of the main island of New Caledonia. It is a 24 ha carbonate islet reaching a maximum of 9 m in elevation. Terrestrial habitats include a central open area with bare ground and patches of various herbaceous plant species surrounded by woody vegetation^78^. Surprise provides refuge for at least 10 breeding seabird species (mostly wedge-tailed shearwater, brown and red-footed booby, great and lesser frigatebirds, black noddy, and sooty tern), totaling *ca*. 5,000 pairs^35^. Reynard islet belongs to the Bampton reefs in the north part of the Chesterfield Islands (central Coral Sea, at *ca*. 550 km north-west from New-Caledonia’s main island). It is a 8.1 ha low-lying carbonate islet, with low and grassy vegetation, housing 8 breeding seabird species for a total of >40,000 breeding pairs^34^. Both islets are surrounded by coral reefs supporting hard coral dominated benthic assemblages^38, 39^. As all islands in the region are hosting seabirds, it was not possible to get control samples at corresponding distances from a seabird-free island. For this reason, a reference station was chosen far from any seabird nesting island and with similar benthic assemblages at 50 m off the reef crest towards the lagoon for both sites (Fig. 1). As a comparison, two sites in the southern lagoon of Nouméa characterized by different levels of anthropogenic impact were sampled: Sainte Marie receiving urban nutrient inputs from the nearby city, and Crouy far from nutrient inputs (15 km from the city, Fig. 1).

### Coral and guano sampling and analysis

Coral collection (sampling license issued by Government of New Caledonia) took place in November 2015 (austral summer), during the breeding season of most of the seabird species present in the study area^34, 35^. The stations were chosen to be gradually farther away from the islets and followed that of a co-occurring and more extensive coral survey, which is why the site distances differed between islets. At Surprise, *P*. *damicornis* was collected by SCUBA at 25, 50 and 100 m from the shore. At Reynard, samples were collected at 50, 400 and 800 m from the shore (Fig. 2). In Nouméa, corals were sampled in December 2014. Five branch portions (5 cm) were cut from five parent colonies at each sampling station (~5 m depth) and frozen until analysis. The tissue was removed from the skeleton using an air-pick^79^ and homogenized with a Potter tissue grinder. Zooxanthellae and coral tissue were separated by centrifugation^80^ and tissue was filtered through pre-combusted 25mm GF/F filters to concentrate coral tissues. Both zooxanthellae and filters were frozen, dried at 60 °C, and encapsulated in tin/silver cups for δ^15^N analysis (zooxanthellae were ground). Guano was also sampled from the nests of different seabird species at Surprise and Reynard islets, dried, homogeneized and prepared as described above for δ^15^N analysis (i.e., the red-footed booby *Sula sula*, the great frigatebird *Fregata minor* and the brown booby *Sula leucogaster plotus*). All samples were analyzed using a Thermo Delta Advantage mass spectrometer in continuous flow mode connected to a Costech Elemental Analyzer via a ConFlo IV at Union College (Schenectady, NY, USA). The combined uncertainty (analytical uncertainty and average correction factor) for δ^15^N (Air) is ±0.15‰, based on an in-house acetanilide standard.

### Nutrient sampling and analysis

Unfiltered triplicates of surface seawater 40 ml samples were collected at Reynard islet (i.e., on November 10^th^ and 22^nd^ 2015) and Surprise islet (October 10^th^ 2016 and March 2^nd^ 2017) at different distances from the islets (Table 1). Nutrients were sampled in both reference sites at 50 m from the reef crest. The reference site for Surprise was also sampled at 400 m from the reef crest, and showed similar NOx values than at 50 m (0.47 vs 0.46 µM). This supports our assumption that any distance from the reef crest at the reference site is representative of waters without seabird influence. Samples were also collected in Nouméa, at Sainte Marie and Crouy, on October 31^st^ 2014. All water samples were fixed with HgCl_2_ pending nitrate + nitrite (NO_3_ + NO_2_) analysis. Nitrate + nitrite (reported as NOx) concentrations were determined according to Raimbault *et al*.^81^ on a Bran-Luebbe III continuous flow autoanalyzer.

### Statistical analyses

The δ^15^N values of coral tissues and zooxanthellae and nutrient concentrations sampled at different distances from the shore were tested for difference against the reference sites with Generalized Linear Models (GLM) in R^82^. For each GLM, the chosen islet reference site (see above) was defined as the model’s intercept, which allowed to directly test for a significant difference in the values observed at each station against that of the reference site for that islet. Due to the small sample size, model performance was evaluated by a visual examination of the distribution of the quantile residuals as implemented in the R package ‘statmod’^83^. A Gamma error distribution with an inverse link was selected for the final models as its properties matched those of the response variables and it resulted in improved residual diagnostics without the need for transformation. A p-value of <0.05 was considered as statistically significant. Reported values throughout were expressed as mean ± SD.

## Electronic supplementary material

Supplementary Tables
