# Supplementary material for: Seabirds supply nitrogen to reef-building corals on remote Pacific islets
Source: Sci Rep. 2017 Jun 16;7:3721. doi: 10.1038/s41598-017-03781-y (PMC5473863; doi:10.1038/s41598-017-03781-y)
Supplement: Supplementary file 1 — Supplementary Tables [file 41598_2017_3781_MOESM1_ESM.pdf]

Seabirds supply nitrogen to reef-building corals on remote Pacific islets

For Scientific Reports

Anne Lorrain[1*], Fanny Houlbrèque[2], Francesca Benzoni[2,3], Lucie Barjon[1], Laura Tremblay-Boyer[5], Christophe Menkes[5], David P. Gillikin[6], Claude Payri[2], Hervé Jourdan[7], Germain Boussarie[2], Anouk Verheyden[6] and Eric Vidal[7]

Supplementary Table S1. Estimated GLM coefficients for the NOx measurements as a function of site, with the reference site defined as the model intercept (Reynard/Surprise islet)

**NOx (Reynard)**	**Distance**	**Estimate**	**Std. Error**	**t value**	**p value**
	Intercept (reference)	7.059	1.219	5.791	0.0004
	50	-6.649	1.221	-5.446	0.0006
	400	-4.455	1.299	-3.429	0.0090
	800	5.186	2.441	2.125	0.0663

**NOx (Surprise)**	**Distance**	**Estimate**	**Std. Error**	**t value**	**p value**
	Intercept (reference)	2.123	0.165	12.889	0.0000
	10	-2.075	0.165	-12.593	0.0000
	25	-2.078	0.165	-12.612	0.0000
	50	-1.761	0.167	-10.541	0.0000
	100	-0.674	0.199	-3.379	0.0055
	400	-0.941	0.289	3.254	0.0069

Supplementary Table S2. Estimated GLM coefficients for coral tissue/zooxanthellae $\delta^{15}N$ measurements as a function of site, with the reference site defined as the model intercept (Reynard/Surprise islet)

**Reynard**	**Coral**	**Distance**	**Estimate**	**Std. Error**	**t value**	**p value**
		Intercept (reference)	0.189	0.009	21.154	0.0000
		50	-0.056	0.01	-5.533	0.0000
		400	0.012	0.011	1.097	0.2872
		800	0.031	0.011	2.785	0.0122
	**Zooxanthellae**	Intercept (reference)	0.201	0.009	22.388	0.0000
		50	-0.065	0.01	-6.278	0.0000
		400	0.019	0.012	1.52	0.1422
		800	0.029	0.012	2.427	0.0235

**Surprise**	**Coral**	**Distance**	**Estimate**	**Std. Error**	**t value**	**p value**
		Intercept (reference)	0.222	0.007	31.549	0.0000
		25	-0.093	0.008	-11.491	0.0000
		50	-0.061	0.008	-7.221	0.0000
		100	-0.047	0.01	-4.739	0.0003
	**Zooxanthellae**	Intercept (reference)	0.246	0.006	41.08	0.0000
		25	-0.117	0.007	-17.281	0.0000
		50	-0.072	0.007	-10.113	0.0000
		100	-0.052	0.009	-6.046	0.0000
